# Achieving net-zero in the dry eye disease care pathway

**DOI:** 10.1038/s41433-023-02814-3

**Published:** 2023-11-13

**Authors:** Samuel G. Latham, Richard L. Williams, Liam M. Grover, Saaeha Rauz

**Affiliations:** 1 Academic Unit of Ophthalmology, Institute of Inflammation and Ageing, College of Medical and Dental Sciences, University of Birmingham, Birmingham, UK; 2 Birmingham and Midland Eye Centre, Sandwell and West Birmingham NHS Trust, Birmingham, UK; 3 School of Chemical Engineering, College of Engineering and Physical Sciences, University of Birmingham, Birmingham, UK; 4 Healthcare Technologies Institute, University of Birmingham, Birmingham, UK

**Keywords:** Corneal diseases, Technology

## Abstract

Climate change is a threat to human health and wellbeing across the world. In recent years, there has been a surge in awareness of this crisis, leading to many countries and organisations setting “net-zero” targets. This entails minimising carbon emissions and neutralising remaining emissions by removing carbon from the atmosphere. At the 2022 *United Nations Climate Change Conference* (COP27), commitments to transition away from fossil fuels and augment climate targets were underwhelming. It is therefore imperative for public and private sector organisations to demonstrate successful implementation of net-zero and set a precedent for the global political consensus. As a top 10 world employer, the United Kingdom *National Health Service* (NHS) has pledged to reach net-zero by 2045. The NHS has already taken positive steps forward, but its scale and complexity as a health system means stakeholders in each of its services must highlight the specifications for further progress. Dry eye disease is a chronic illness with an estimated global prevalence of 29.5% and an environmentally damaging care pathway. Moreover, environmental damage is a known aggravator of dry eye disease. Worldwide management of this illness generates copious amounts of non-recyclable waste, utilises inefficient supply chains and involves recurrent follow-up appointments and prescriptions. By mapping the dry eye disease care pathway to environmental impact, in this review we will highlight seven key areas in which reduced emissions and pollution could be targeted. Examining these approaches for improved environmental sustainability is critical in driving the transformation needed to preserve our health and wellbeing.

## Abstract (zh)

气候变化对全世界人类健康和福祉构成威胁。近年来, 人们对这场危机的认识显著深入增, 因此许多国家和组织设定了“净零”目标。为达目标需要尽量减少碳排放, 并通过消除大气中的碳来中和剩余排放。在2022年联合国气候变化大会(COP27)上, 关于减少化石燃料使用和加强气候管理的承诺未达目标。因此, 公共和私营部门组织必须证明净零排放的成功实施, 并为全球政治共识开创先例。作为世界十大雇主之一, 英国国家卫生服务局(NHS)承诺在2045年实现净零排放。NHS已采取积极行动, 但卫生系统的规模和复杂性意味着每一项服务的获益者必须强调进一步开展的规范性。干眼症是一种慢性疾病, 估计全球患病率为29.5%, 是受环境影响的一种疾病。此外, 环境破坏是已知的干眼症的加重因素。在世界范围内对这种疾病的治疗产生了大量不可回收的环境污染物, 使得供应链利用低效, 其中包括反复的复诊预约单和处方的资源浪费。通过绘制环境对于干眼护理路径影响的关系图谱, 在本文中, 我们将强调可能以减少排放和污染为目标的7个重要领域, 研究这些方法对于改善环境可持续性和推动保护我们健康与福祉所需的转型至关重要。

## Introduction

Environmental pollution is increasingly endangering human health and wellbeing by provoking extreme weather events, damaging ecosystems, facilitating spread of infectious diseases, exacerbating socioeconomic pressures and destabilising food and water supplies [1]. Growing recognition of this worldwide crisis is prompting countries and organisations to set “net-zero” targets. The United Nations defines net-zero as cutting greenhouse gas emissions to as close to zero as possible, with any remaining emissions re-absorbed from the atmosphere [2]. In 2022, the United Kingdom (UK) National Health Service (NHS) became the world’s first health system to embed net-zero into legislation [3]. Published strategies to lower emissions controlled by the NHS include renewable energy and heat generation, electrification of the transport fleet, reduced usage of plastics and increased digitisation [4]. Lowering emissions influenced by the NHS is more complicated and it is estimated this will take an additional five years to reach net-zero [4]. The key targets here are the NHS supply chain and travel of patients, staff and visitors, which constitute approximately 62% and 10% of overall emissions respectively [4]. Each of the four health systems within the NHS has pledged to reach net-zero for the emissions it controls and can influence by 2045 [5].

Ophthalmology represents 10% (9 million annually) of NHS outpatient appointments and 6% of all surgeries in the UK: demand for ophthalmology services is forecasted to surge by 40% over the next 20 years [6]. The environmental damage caused by ophthalmology services has generated considerable interest particularly relating to the Carbon footprint created by surgical operations [7–18]. This includes cataract surgeries performed in the UK. These produce substantially more greenhouse gases and non-recyclable waste than in other countries; primarily due to more frequent procurement of disposable equipment [19, 20].

There is little published data evaluating the impact of chronic eye pathologies that are medically managed on environmental sustainability.

A review of large international epidemiology studies has shown dry eye disease affects between 5% and 50% of the adult population worldwide [21]. Recent estimates from a Bayesian approach indicate a 29.5% global prevalence of dry eye disease that satisfies the Tear Film and Ocular Surface Society’s Dry Eye Workshop (TFOS DEWS II) diagnostic criteria [22]. Moreover, the TFOS Lifestyle Report provides extensive evidence that environmental damage is an aggravator of dry eye disease, leading to a vicious cycle [23]. By 2032, the global dry eye disease treatment market will likely be valued at over £7 billion [24]. Many dry eye disease patients must apply eye drops frequently for the rest of their lives, with increased administration frequencies associated with poorer psychological outcomes [25]. Management of dry eye generates copious amounts of non-recyclable waste, utilises inefficient low-temperature controlled supply chains (cold chains) and involves frequent follow-up appointments, prescriptions and travelling. With the net-zero goals of the NHS in mind, there is a clear need for affective innovation in this care pathway and the surrounding supply chains. By mapping the dry eye disease care pathway to environmental impact, we will model areas in which reduced emissions and pollution could be targeted to restore planetary ecosystems and ocular surface health. Opportunities and limitations for improved environmental sustainability with an emphasis on each domain’s pivotal role in driving carbon emission transformation is explored.

## The NHS dry eye disease care pathway

**Fig. 1:**
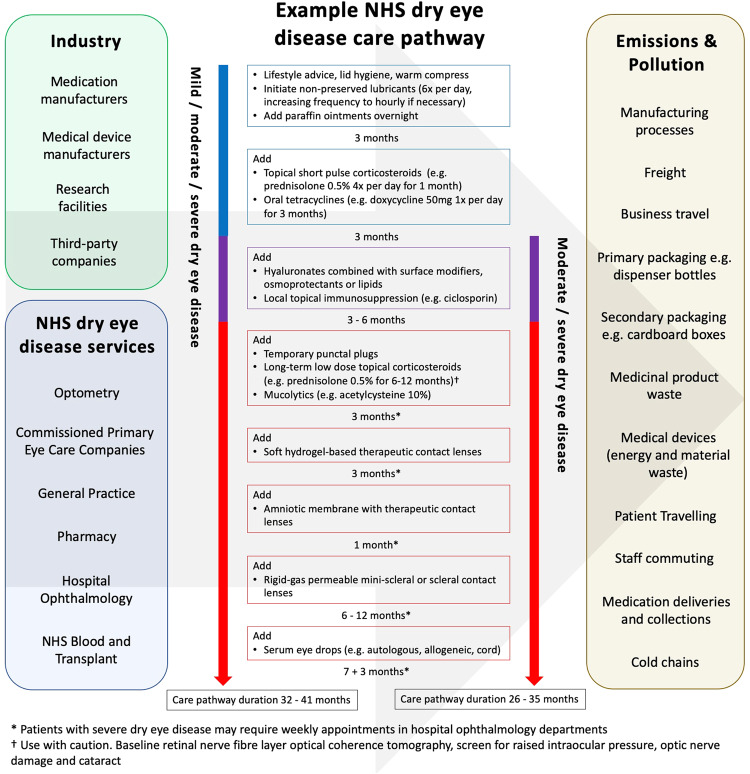
Current landscape of the NHS dry eye disease care pathway.

Each patient follows a personalised journey along the dry eye disease care pathway tailored to disease severity, underlying pathophysiology, therapeutic success rates, locally available dry eye disease services, professional opinions, waiting lists and patient choice. Achieving good adherence to treatments in dry eye disease is a well-recognised challenge and also impacts the patient journey along this care pathway [26]. Figure 1 provides an example NHS dry eye disease care pathway and illustrates the frequency of appointments and multiple treatment trials to optimise patients’ symptoms and signs. It also highlights the different industrial and NHS organisations involved and the varying sources of emissions and pollution that subsequently occur. The chronicity of dry eye disease and short therapeutic durations of medications means that patients require large volumes of medications during their lifetime, generating substantial amounts of material waste. This waste comprises primary and secondary packaging of medications as well as medicinal products themselves. In addition, the multifactorial pathophysiology and fluctuating severity of dry eye disease necessitates several therapeutic-trial-periods and frequent ophthalmology follow-up appointments (sometimes weekly in the severest of cases). These characteristics enforce further material waste and greenhouse gas emissions, particularly when patients travel by car to appointments and to collect prescriptions.

## Recent innovations and future opportunities

### Replacing single dose plastic vials

**Fig. 2:**
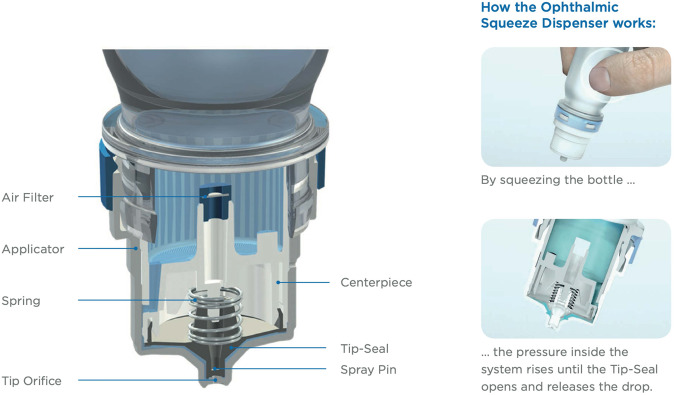
Aptar’s Ophthalmic Squeeze Dispenser Tip-Seal technology. Image courtesy of Aptar Pharma.

Packaging of medications can be divided into primary packaging and secondary packaging. Primary packaging houses the medication and includes blister packs and dispenser bottles. Secondary packaging typically comprises cardboard boxes and paper instructions. Eye drops are either preserved (containing preservatives) or preservative-free. Historically, all preservative-free eye drops were dispensed using single dose eye drop vials as multidose dispensers were unable to maintain adequate sterility of these solutions. The Ophthalmic Squeeze Dispenser (AptarGroup; Crystal Lake, Illinois, USA), Novelia® (Nemera; La Verpillière, France), COMOD® (URSAPHARM Arzneimittel; Saarbrücken, Germany), ABAK® (Théa Laboratories; Clermont-Ferrand, France) and 3K® (Aero Pump; Hochheim am Main, Germany) are all examples of currently available innovative multidose preservative-free systems. These dispensers possess their own unique features to prevent contamination and ensure safe delivery of preservative-free lubricating eye drops (Fig. 2). Aptar’s Ophthalmic Squeeze Dispenser is currently the only multidose preservative-free system approved (as part of a medicinal product seeking approval at the time) by the UK’s Medicines and Healthcare products Regulatory Agency (MHRA) and the U.S. Food and Drug Administration (FDA) to deliver metered doses of topical medications.

**Fig. 3:**
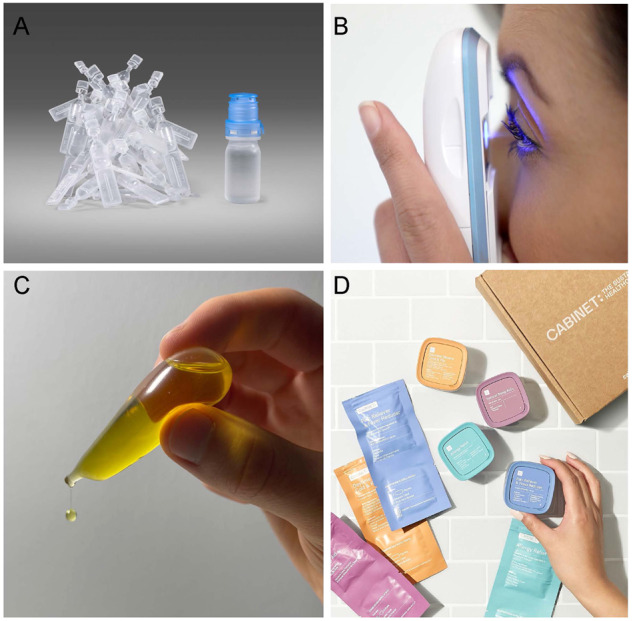
Existing and potential dispensing methods for dry eye disease therapeutics. **A** Blow-fill-seal single dose vials compared with Aptar’s Ophthalmic Squeeze Dispenser showing the number of unit dose equivalents to the single multidose bottle (image courtesy of Aptar Pharma); **B** Eyenovia’s Optejet; **C** The Notpla Pipette; **D** Cabinet Health’s refillable medicine bottles and fully compostable refill pouches.

The decision to recommend eye drops in single dose vials or multidose dispensers is multifactorial and should account for patient preference, which can be affected by manual dexterity limitations such as age and underlying health conditions e.g., arthritic hands. Single dose eye drops vials use around eight times more plastic (Fig. 3A) and nine times more energy for transportation compared to their multidose counterparts [27]. Furthermore, the use of multidose bottles eliminates product waste as they can be used until the last drop [27]. Assuming that 200 million people globally use artificial tears or lubricants four times per day, use of single dose vials exclusively would create an additional 22 600 tonnes of plastics disposal per month [28]. Under the same assumption, whilst recognising the typical disposal volume of medication in a 0.3 ml single dose vial is 140 μl, use of single dose vials exclusively would also lead to 3,360,000 litres of medication waste [29].

Alternative methods of ocular surface drug delivery, which in the future might lessen the need for plastic dispensers, include punctum plug drug delivery systems, ophthalmic inserts and microdosing spray dispensers such as the Optejet® (Eyenovia; New York City, New York, USA) (Fig. 3B). In patients where the use of single dose dispensers is needed for safety reasons, introduction of biodegradable plastics may be a better strategy for minimising environmental pollution. Notpla (London, UK) is a sustainable packaging company and an Earthshot Prize winner 2022 that manufactures 100% biodegradable plastics from seaweed and plants [30]. Amongst its products is the Notpla Pipette, which provides controlled pouring of single-use edible oils (Fig. 3C). Adaptation of products like the Notpla Pipette to create 100% biodegradable single dose eye drops vials is a feasible and potentially revolutionary goal. In years to come, biodegradable plastic-substitutes might also be utilised elsewhere in the dry eye disease care pathway, such as the primary packaging for oral medicines. Cabinet Health (New York City, New York, USA) is a company already selling refillable medicine bottles and 100% compostable medicine refill pouches made from wood cellulose and biosealants (Fig. 3D). It is reasonable to suggest this concept could soon be featured in dry eye disease management.

### Recycling

Despite the existing ability to recycle the types of plastics used in primary packaging of dry eye disease treatments, most of the plastic will end up in landfill or an incinerator [28]. This is because the separation of recyclable plastics at sorting facilities will prioritise larger items, leaving smaller items to ‘slip through the net’ [28]. In addition, many of the multidose eye drops dispensers contain metallic valves and springs in their nozzles, which would need to be separated [30]. This issue is consolidated by the fact it is cheaper to manufacture new plastics than it is to collect, sort and recycle them [31]. TerraCycle (Trenton, New Jersey, USA) is a company overcoming this problem. As part of their innovative business model, TerraCycle is able to recycle more challenging-to-recycle materials with additional funding received from brands that wish to promote environmental sustainability [32]. In the United States, TerraCycle has partnered with Bausch + Lomb (Quebec, Canada). Together they have launched the Biotrue® Eye Care Free Recycling Program, which enables citizens to recycle the primary packaging of contact lenses and eye drops. Similar initiatives have been established in the UK by Acuvue (Jacksonville, Florida, USA), Vision Direct (London, UK) and Spescavers (Guernsey, UK), but these are limited to recycling the primary packaging of contact lenses only. TerraCycle has also established the Empty Medicine Blister Packs - Zero Waste Box™ scheme whereby UK pharmacies purchase a recycling bin, which TerraCycle collects and processes. Encouragingly, the financial cost incurred by pharmacies as part of this scheme is reportedly offset by an increase in customer footfall [33]. A comparable scheme has been introduced by Superdrug (Croydon, UK). Recycling the containers of topical ointments used in dry eye disease imposes an additional challenge. Many of these ointments are packaged in plasticised, lacquered aluminium tubes closed with tamper evident polyethylene caps. In general, these tubes are not recyclable as the plastic and aluminium layers are too burdensome to separate [34]. Recent development of similar, yet fully recyclable tubes offer more environmentally sustainable alternatives for ointment dispensing in dry eye disease (Table 1). Many of these tubes are made from already recycled materials, thus contribute to a circular economy. Tubes made purely from aluminium possess advantages such as infinite recyclability and favourable cost-benefit separation at recycling facilities. Furthermore, recycling aluminium, requires 95% less energy than its primary production from ore [35].

**Table 1:** Examples of eco-friendly tubing suitable for pharmaceutical use.

Company	Product	Composition	Recyclability
**Hoffmann Neopac** (Thun, Switzerland)	Polyfoil® Mono Material Barrier tube	High-density polyethylene	>95% recyclable
**Emballator** (Bradford, UK)	100% Post-Consumer Recycled aluminium tube	100% Post-Consumer Recycled aluminium	100% recyclable
**Alltub Group** (Boulogne-Billancourt, France)	Green Tube	>95% Post-Consumer Recycled aluminium	100% recyclable
**Tubex** (Rangendingen, Germany)	The Blue Tube®	100% post-industrial aluminium	100% recyclable
	The Blue Tube Evo®	>95% consumer recycled aluminium	100% recyclable
**Tubapack;** (Žiar nad Hronom, Slovakia)	Go Green! Tube	100% Post-Consumer Recycled aluminium	100% recyclable
**Lisson Packaging** (Taihe, China)	Aluminium Collapsible Tubes	99.7% high purity aluminium slugs	100% recyclable

In terms of secondary packaging, general guidance for UK citizens suggests that cardboard boxes and any paper inserts can be domestically recycled [36]. Nevertheless, it is worth noting that some cardboards are easier to recycle than others. Cardboards that are laminated in plastics such as polyethylene, to provide a moisture barrier and structural integrity, make recycling more difficult [37]. Additional recycling processes are required to separate the materials and subsequently incineration or landfill may result instead [37]. Therefore, potentially recyclable cardboard is being wasted and further unnecessary pollution of plastics is taking place. Inspection of eye drops secondary packaging reveals a clear lack of recycling instructions. This suggests that secondary packaging of dry eye disease medications might often be made from these difficult-to-recycle cardboards. Potential alternatives for secondary packaging include cardboards with innovative eco-friendly coatings (Table 2**)**. These coatings are able to maintain waterproof barriers and structural stability whilst being easily recyclable, biodegradable and cost-comparable.

**Table 2:** Examples of eco-friendly coatings for potential use in secondary packaging.

Company or institute	Product	Composition	Features
**GWP Group** (Swindon, UK)	Liquiguard	Blended waterborne acrylic lattice	Approved for food contact, fully biodegradable and fully recyclable
**Cortec Corporation** (Saint Paul, Minnesota, USA)	EcoShield®	Acrylic resin-based	Approved for food contact and fully recyclable
**LiquiGuard Technologies** (Fort Lauderdale, Florida, USA)	CorruCote	Water borne acrylics and styrennated acrylics	Approved for food contact and easily recycled
**Papkot** (Strasbourg, France)	Papkot™	Ceramic	Approved for food contact, fully biodegradable and 99.99% recyclable
**The University of Tokyo** (Bunkyō, Japan)	Choetsu	Methyltrimethoxysilane, isopropyl alcohol and tetraisopropyl titanate	Fully biodegradable and dirt- and bacteria-repelling
**Notpla** (London, UK)	Notpla Coating	Seaweed	Approved for food contact and fully compostable

Although not in the traditional fashion of recycling, “recycling” in dry eye disease may also apply to blood products that would otherwise be wasted. New-born’s blood can be collected from the placenta via the umbilical cord and then pooled. This cord blood is a valuable resource for hematopoietic transplantation. When the cord blood is not suitable for hematopoietic transplantation, a strategy called Multicomponent Cord Blood Banking may be used to develop serum eye drops for the treatment of dry eye disease [38]. This strategy has been developed in Italy and Spain and could provide an additional source for serum eye drops in the UK. If cord blood was collected and processed efficiently, this could potentially reduce the Carbon footprint of existing serum eye drops manufacturing.

### Diminishing cold chains

**Fig. 4:**
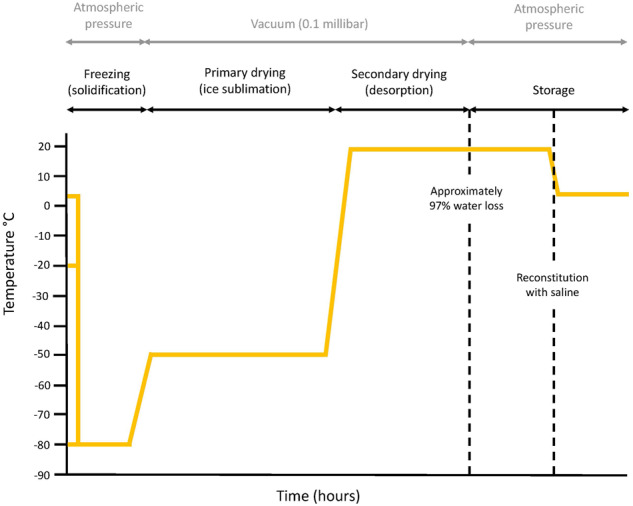
Example schematic illustration of serum eye drops lyophilization.

Patients chilling eye drops in their usual domestic fridge or freezer is not harmful towards the environment. Contrastingly, it is harmful when eye drops require refrigeration or freezing during manufacturing and transportation. It is also problematic when patients require an additional fridge or freezer specifically for storing their eye drops. In the UK, serum eye drops are supplied as an unlicensed medication by *NHS Blood and Transplant* (NHSBT). Storage temperature of serum at NHSBT is –80 °C. Once processed, the serum eye drops are transported and stored for up to twelve months at –20 °C to preserve epitheliotropic factors and minimise the risk of microbial proliferation [39]. An alternative method of preventing microbial contamination of serum eye drops, which could lessen the need for freezing is photochemical pathogen inactivation. Preclinical study data from an animal-model demonstrates restoration of corneal epithelium with the use of solvent-detergent treated serum eye drops [40]. These data support the possibility of adopting photochemical pathogen inactivation in human serum eye drops. Another way of potentially lessening cold chains is lyophilization or freeze drying. This is the process of removing water from a (typically perishable) material at low temperature to transform it to a more stable solid state so it can be stored safely at a higher temperature (Fig. 4). The desired effect is enhanced material stability and shelf life, eliminating dependence on cold chains. In serum eye drops, the use of lyoprotectants such as trehalose can be added to protect the proteins from freezing related stress. Preclinical studies have shown that lyophilized serum eye drops maintain their biochemical properties and have comparable therapeutic effects to fresh serum [41, 42].

### Alternative therapies

There have been recent developments in devices that treat meibomian gland dysfunction, which is present in approximately 86% of dry eye disease patients [43]. New devices such as Lipiflow® (Johnson & Johnson Vision; Jacksonville, Florida, USA), TearCare® (Sight Sciences; Menlo Park, California, USA), EyeXPress™ (Holbar Medical Products; Tyler, Texas, USA), Tixel® (Novoxel; Netanya, Israel), MiBo Thermoflo® (Mibo Medical Group; Dallas, Texas, USA), iLux2® (Alcon; Geneva, Switzerland), E>Eye® (E-SWIN; Houdan, France), OptiLight™ and M22 Optima™ IPL (Lumenis; Yokneam Illit, Israel) utilise thermal or intense light pulsation systems to restore meibomian gland function and release oils around the ocular surface. Therapeutic success with these devices can reduce reliance on medications and may subsequently lower Carbon emissions. Electricity-reliant therapies like these also provide an opportunity to employ renewable energy. This same opportunity applies to BlephEx™ (Blephex LLC; Brentwood, Tennessee, USA), Blephasteam® (Théa Laboratories) and NuLids™ (NuSight Medical; Escondido, California, USA) to treat blepharitis and neurostimulation therapy such as TrueTear® (AbbVie; North Chicago, Illinois, USA) to treat lacrimal gland dysfunction. Moreover, a patent filed by Oculeve (AbbVie; North Chicago, Illinois, USA) highlights the possibility of integrating electrical stimulation of lacrimation technology into contact lenses [44]. Further alternative therapies that may reduce reliance on eye drops include meibomian gland probing, mesenchymal stem cell injection into the lacrimal gland and Tyrvaya® (varenicline solution) nasal spray (Oyster Point Pharma; Princeton, New Jersey, USA). The therapeutic mechanisms that underpin each of these treatments will no doubt be explored further, leading to the introduction of new treatments in the coming years.

### Longer lasting eye drops

The eye drops market continuously evolves by welcoming increasingly sophisticated active ingredients that reduce ocular surface inflammation and tear film instability. With net-zero ambitions in mind, improved environmental sustainability can be achieved through eye drops that exhibit prolonged ocular surface retention; thus reducing administration quantities and medication-associated waste. Artificial tears provide only a transient therapeutic affect as they are rapidly displaced from the ocular surface by tear drainage, absorption and blinking [45]. Enhancing the viscosity of eye drops is a well-recognised strategy for prolonging ocular surface retention and excipients for this include carbomer 940 (polyacrylic acid), carboxymethyl cellulose, dextran, hyaluronic acid, HP-guar, hydroxypropyl methylcellulose, polyvinyl alcohol, polyvinylpyrrolidone and polyethylene glycol [46]. The future development of novel excipients will perhaps also incorporate both mucoadhesive and drug release controlling properties like those in thiolated cyclodextrins [47]. Another strategy for prolonging retention is restoring the tear film’s lipid layer by incorporating oils into eye drop solutions. A noteworthy development in this strategy is the use of cationic submicron oil-in-water vectors containing positively charged oil nanodroplets. When instilled, these positively charged oil nanodroplets are electrostatically attracted to the negatively charged mucin layer of the tear film, resulting in improved ocular surface retention time [48]. Adhesion to the mucin layer can also be achieved through excipients that promote hydrogen bonding, disulfide bonding and interpenetration of mucoadhesive polymers and chain entanglements [49]. Nanocarrier systems such as nanoliposomes and nanoparticles offer prolonged ocular surface retention either by mimicking the lipid components of the tear film or through displaying hydrophilic or mucoadhesive properties [50]. An area of evolving research is the use of biomaterials such as hydrogels to increase the longevity of symptom relief in dry eye disease. Hydrogels are organic, hydrophilic and viscoelastic polymeric networks that provide structural support and protection for some of the most delicate tissues in the human body [51]. Both biopolymer and synthetic hydrogels are versatile lubricating substances developed through chemical or physical cross-linking and have demonstrated prolonged ocular surface retention as well as excellent biocompatibility and biodegradability [52–54]. Ocular surface retention time of hydrogels can be further enhanced by manipulating their physical and chemical structures so they are sensitive to environmental changes such as temperature, pH and ionic strength [55–57]. It is noteworthy that the cross-linked matrix within hydrogels can act as vehicles for drugs and allows increased drug retention time on the ocular surface [58, 59]. Furthermore, hydrogels are commonly used in the manufacturing of contact lenses, which also offer prolonged retention of eye drops and sustained release of ocular surface drugs [60, 61].

### Reducing travelling

**Fig. 5:**
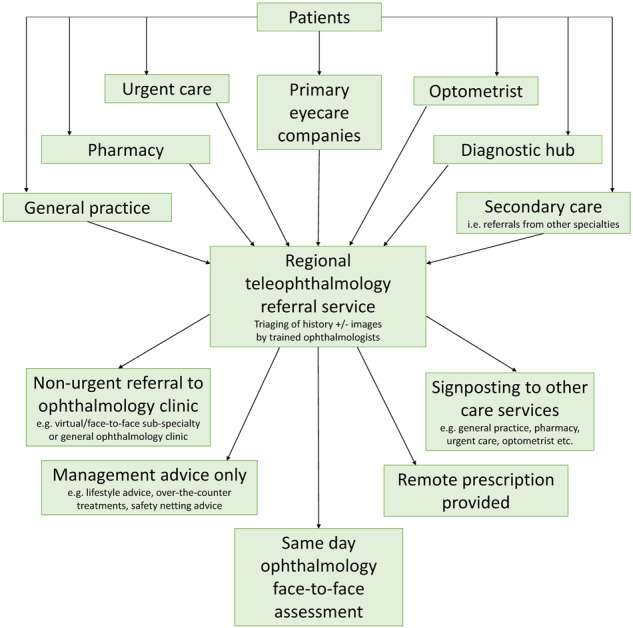
Flow-diagram illustrating the potential use of regional teleophthalmology services to avoid inappropriate referrals and unnecessary patient travelling.

NHS England aims to achieve eye care closer to home by optimising the community optometry workforce, commissioning of services via Primary Eyecare Companies and establishing diagnostic and treatment hubs [62]. Filtering schemes to safely reduce the number of inappropriate referrals to NHS outpatient ophthalmology clinics is a target area, which has already shown to be effective [63, 64]. Teleophthalmology serves as an innovative measure in reducing travelling and carbon footprint, and its use has been accelerated by the recent SARS-Cov-2 pandemic [65, 66]. Currently, the principal use of teleophthalmology in the NHS involves patients attending their local optometric practice where the use of various imaging and video systems allow capturing of key examination features [67]. These images or videos are sent to the nearest ophthalmology department where management decisions are made [67]. A flow-diagram illustrating what teleophthalmology might resemble in the future is shown in Fig. 5. Guidance for virtual consultations is already available and could become embedded into teleophthalmology triaging [68]. Evolution of teleophthalmology to adequately address dry eye disease may require use of traditional diagnostics like Schirmer’s test or fluorescein break-up time, or adoption of new technologies like InflammaDry® (Quidel; San Diego, California, USA), TearLab® Osmolarity System (TearLab; Escondido, California, USA), Keratograph® 5M (Oculus Optikgeräte; Wetzlar, Germany), Idra ocular surface analyzer (SBM Systemi; Turin, Italy) LipiScan® and LipiView II® (Johnson & Johnson Vision Care; Jacksonville FL, USA) at Primary Eyecare Companies and optometric practices. The end goal of teleophthalmology is to enable safe and reliable assessment of patients from their own home. The smartphone app DryEyeRhythm is an example of how multivariate-adjusted logistic regression analysis can be used to identify risk factors for symptomatic dry eye and to identify risk factors for undiagnosed symptomatic dry eye [69]. The combination of growingly sophisticated home devices and artificial intelligence algorithms instils optimism.

**Fig. 6:**
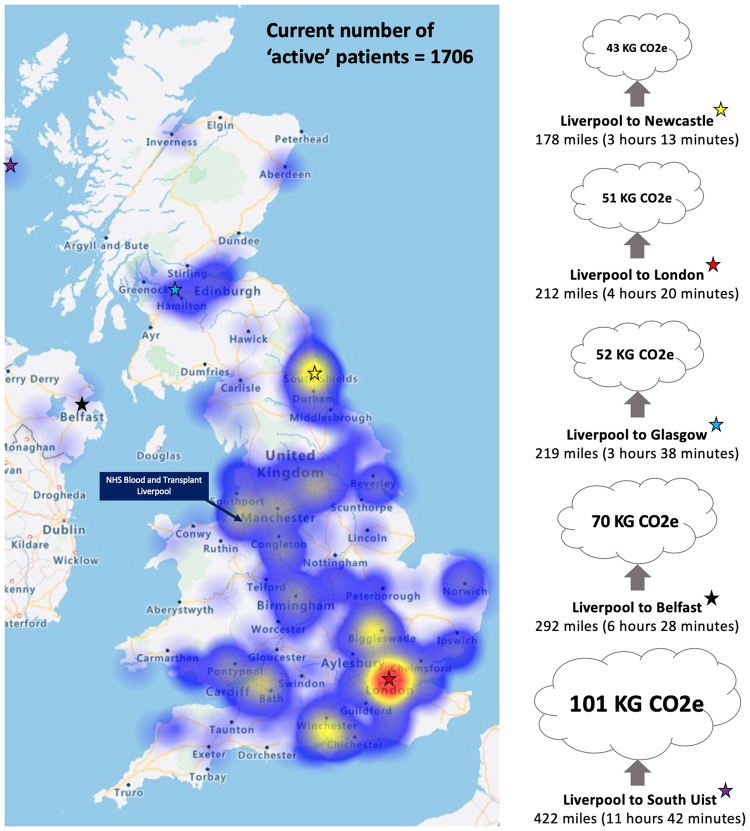
Heat map of residential postcodes for NHSBT serum eye drop users referred in 2022 with estimates for CO_2_ equivalent emissions per delivery (one-way). Original image courtesy of NHSBT Serum eye drops service.

Pharmacies also have a significant role to play in reducing travelling associated emissions. Numerous UK-based pharmacy companies such as Kamsons Pharmacy (Uckfield, UK), Avicenna (Croydon, UK) and the Pharmacy Group (Leeds, UK) have begun integrating electric vehicles for medication deliveries [70]. Furthermore, growing numbers of pharmacies are installing Pro Delivery Manager (Machynlleth, UK), which is a digital system that optimises delivery routes and cuts delivery van fuel consumption by a reported 12% [70]. The introduction of electric vehicles and delivery management systems could lower emissions from other dry eye disease related services such as the collection of blood and delivery of serum eye drops by NHSBT. The large majority of serum eye drops provided by NHSBT are allogenic and produced from blood collected at donation centres near the centralised NHSBT serum eye drops processing facility in Liverpool. However, patients who use autologous serum eye drops sometimes travel long distances to donate their own blood at specific apheresis centres. For example, the nearest collection centre for patients living in South-West England is located in Bristol, a distance of up to 200 miles. It is noteworthy that blood donation for the production of autologous serum eye drops takes place in England only. Patients in Wales can travel to donation centres in England, but patients in Scotland and Northern Ireland are offered the option of allogenic serum eye drops alone. All blood destined to become serum eye drops (allogenic and autologous) is transported to the centralised NHSBT serum eye drops processing facility in Liverpool. Following processing, couriers travel to deliver serum eye drops to patients across the UK by car or van and on rare occasions ferry, largely on a per patient basis (Fig. 6). It is possible to combine deliveries if there are two patients who reside in the same area, but this is not a regular occurrence. NHSBT should aim to reduce its Carbon footprint through combining more deliveries. Furthermore, expanding the NHSBT serum eye drops service to include more processing and donation facilities or local stock holding distribution facilities could also help to significantly reduce emissions.

### Promoting sustainability

The UK Government has a large responsibility to fund NHS net-zero. The Small Business Research Initiation (SBRI) Healthcare is an NHS England programme, which runs an annual clinical innovation competition titled Delivering a net-zero NHS. This allows innovations that enhance environmental sustainability to receive financial support. Similar funding is available through the Accelerated Access Collaborative. Academic research into environmentally sustainable healthcare is also receiving financial backing via the National Institute for Health Research and Medical Research Council. In addition, The UK Government is instating regulatory measures to ensure NHS suppliers reduce their Carbon emissions. From April 2027, all NHS England suppliers must publicly report their direct and indirect emissions and release a sustainability plan in line with NHS net-zero. From 2030, suppliers will only qualify for NHS England contracts if they can demonstrate progression towards sustainability. One method for evidencing environmental sustainability is offsetting emissions either directly or via third-party organisations. Offsetting Carbon emissions is not the long-term solution for achieving net-zero but it is a commendable gesture whilst more sustainable practices are being implemented. Education and awareness are also indispensable factors in achieving net-zero [71]. Universities are now integrating innovation and environmentally sustainable healthcare into their curriculums [72, 73]. Meanwhile, NHS Trusts and governing bodies are encouraging multi-disciplinary workers to engage in teaching about eco-friendly healthcare [73–75]. Educating patients on this topic is perhaps more challenging. This could include displaying information in waiting areas and editing existing patient information leaflets to include information about environmental sustainability. Thereafter, increased patient and public involvement can be achieved through interactive net-zero research and fundraisers. Public engagement will also be necessary so the general public is aware of progress and can offer new ideas.

## Current limitations

### Replacing single dose plastic vials

**Fig. 7:**
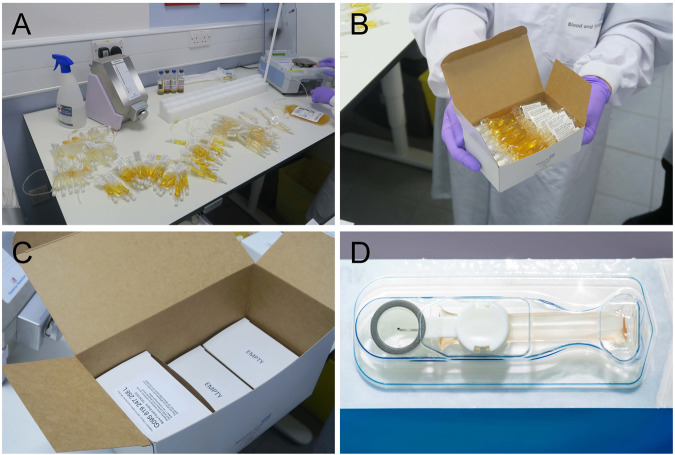
Current packaging for serum eye drops. **A** Meise Medizintechnik closed filling system at NHSBT Liverpool; **B** Serum eye drops vials ready to be frozen and then delivered on dry ice via personal courier; **C** Empty cardboard boxes used as spacers during delivery; **D** mu-Drop micro eye-drops packaging.

Single dose plastic vials are the gold standard in aseptic preservative free eye drops dispensing. They are aseptically manufactured using the blow-fill-seal process and remain sealed until the patient needs them [76]. To prevent contamination in multidose bottles, manufacturers commonly integrate preservatives into the eye drops solution. However, it is well known the use of preservatives in eye drops can cause irritation to the surface of the eye. Multidose preservative-free drops must integrate innovative designs in their tips to achieve one-directional movement of eye drops and prevent contamination and this makes the manufacturing process more expensive [77]. Furthermore, the risk of contamination remains higher in multidose bottles despite their innovative designs [78–80]. The risk of contamination is also increased in punctum plug drug delivery systems and ophthalmic inserts, which often cause irritation and foreign body sensation as well [81, 82]. It is not possible to completely eliminate the risk of contamination with drug delivery. Nevertheless, it is crucial to recognise that some patients are higher risk than others. Regarding serum eye drops, it is vitally important to minimise the risk of contamination, as patients who receive these drops have more severe dry eye disease and are at higher risk of developing infections. The current closed filling system at NHSBT enables aseptic dispensing of serum (Fig. 7A). A potential alternative system is the micro eye drop (mu-Drop; Apeldoorn, Netherlands). This system dispenses serum eyedrops as small as 7 microlitres, thus avoiding reflex tearing upon administration and reducing medication waste and wash-out of serum [83]. However, it employs multi-material single use dispensers in individual blisters and possibly produces even more non-recyclable packaging (Fig. 7D). Replacing single dose plastic vials with multidose preservative-free bottles, alternative devices or biodegradable single dose vials has great potential to benefit the environment. Yet such changes present several regulatory and operational challenges. Container systems alone are subject to regulatory assessment as part of a finished product and substantial validation data on container compatibility with the contents, container robustness and safety with respect to consistent dose release is often presented (or at least known to regulators). Modifying an existing commercially available container may require revalidation at considerable cost and time to the packaging manufacturer and potentially the manufacturer of the finished therapeutic product before launch on the market. Substantial changes, such as a change to a novel material in the construction of the container, may involve the development of a brand-new container by the packaging manufacturer and the business case to support such a change needs to be compelling. These considerations extend to changes to the design of manufacturing processes for products intended for human use. Manufacturing activities are typically governed by Good Manufacturing Practice (GMP) for medicinal products and ISO13485 for medical devices and the process for designing, implementing, and validating process changes can take up significant personnel time, cost and potentially temporary shutdown of a facility.

### Recycling

Whether or not something is municipally recyclable depends on regional recycling companies and their ability to profit from recycling it. If the cost of collecting, sorting and recycling the material is greater than the value of the end product plus subsidies, the material will unlikely be recycled [84]. There are two main strategies for avoiding this problem. The first is improving recycling processes so that more difficult-to-recycle materials are recycled. TerraCycle® is a relevant example mentioned above. However, a key factor impeding the uptake of TerraCycle® is the additional effort required by patients to perform the multiple steps involved. Insufficient awareness of TerraCycle® is also a barrier to entry for both patients and potential business partners. The other main strategy for avoiding unnecessary pollution is making the material waste more recyclable or biodegradable. The issue with primary packaging of dry eye medications is that it must remain small and robust to enable convenient and safe use throughout the day. With secondary packaging, alteration of manufacturing processes to incorporate more eco-friendly cardboards will inevitably incur costs. Evidently, many pharmaceutical companies lack the incentive to make secondary packaging clearly recyclable. There is also a lack of awareness amongst the general public regarding the recyclability of different cardboards. With regards to alternative therapies for dry eye disease, these currently involve the use of non-recyclable plastics such as tape, instrument tips and sheaths. Recognition of this must be heightened so that device manufacturers are encouraged to incorporate more sustainable materials into future models.

With regards to introducing Multicomponent Cord Blood Banking into the manufacturing of serum eye drops, it would be very difficult to achieve a resulting Carbon footprint reduction. This is because cord blood donations are one-off donations of much lower volumes than a standard blood donation. Therefore, more emissions and packaging will be required for collection and processing. The implementation of Multicomponent Cord Blood Banking in Italy and Spain is mostly due to regulatory issues with providing allogenic serum eye drops.

### Diminishing cold chains

Producers of serum eye drops specify shelf lives ranging from 3 to 12 months when frozen and up to 24 hours to 1 week after thawing [85]. The lack of consensus is partly due to internationally varying regulations [86]. In the UK, patients receive a 3–5 months supply of serum eye drops for storage in a domestic freezer [39]. These patients are advised to store the currently used bottle at 4 °C for up to 24 hours. Such practices are supported by studies which suggest epitheliotropic factors are preserved at −20 °C for 6 months and substance P and calcitonin gene-related peptides significantly degrade at 4 °C within 24 hours [87–90]. However, varying results and recommendations can be found within the literature [91]. Current UK practices appear to offer satisfactory safety and efficacy whilst also avoiding overburdening frequencies of blood donations and serum eye drops deliveries. Although cold storage of serum eye drops is potentially harmful for the environment, it is vitally important to minimise the risk of contamination and maintain efficacy.

### Alternative therapies

Most of the devices and services are not provided by the NHS and are expensive alternatives to conventional treatment. There is also limited evidence to suggest these treatments are superior to the conventional ones provided by the NHS. If the manufacturers and providers of these alternative therapies are able to lower the costs, more patients will receive the treatments and better comparisons of efficacy can be made. Conventional treatments may also be preferred because the majority of patients can independently administer them when desired.

### Longer lasting eye drops

Increasing the viscosity of eye drops enables longer residence time on the ocular surface but can create unwanted affects such as eyelash debris, discomfort and blurred vision [92]. Therefore, the ideal eye drops formulation should have alternating viscosities. To avoid rapid displacement of eye drops, the formulation should be viscous when the eye is open [93]. To avoid friction, discomfort and uneven spread of eye drops the formulation should be less viscous during blinking [94–96]. This combination is known as shear-thinning behaviour and is displayed in solutions containing excipients such as hyaluronic acid [97]. Viscosity-effecting factors such as temperature and pH changes upon contact with the ocular surface must be taken into account. There are also eye drops constituents that can affect the behaviour of formulations such as ocular surface nutrients and anti-inflammatory drugs. Excipients can provide advantageous physical and chemical properties for eye drops. However, the number of accepted excipients for eye drops is limited by the delicateness of ocular tissues. It is therefore challenging to produce a formulation that satisfies the needs of patients with symptomatic dry eye disease. In addition, some of the eye drops that address the underlying pathophysiology of dry eye disease can take 3–6 months to work, which impedes their acceptability [98]. These issues are compounded by a lack of head-to-head comparison studies between various eye drops in dry eye disease. Future studies are needed to evaluate the therapeutic duration of different eye drops in varying subgroups.

### Reducing travelling

More local delivery of care for dry eye disease requires radical restructuring of the care pathway. Challenges will include publishing new guidance, upskilling community healthcare providers, ensuring safe handling of workload capacity, investing in new equipment and establishing correct flow of finances. Already, there is an existing issue of inconsistent services provided by community optometrists. This is due to the disparate activity of clinical commissioning groups across the UK, which leads to a postcode lottery of optometry services [99]. Another issue is the disconnection of electronic systems between community optometric practices, GP practices and NHS hospitals [100]. Improvement in this area is essential for more local delivery of care. Integration of teleophthalmology also presents numerous challenges. The most apparent difficulty is the willingness of stakeholders to deviate from face-to-face appointments in which a patient-doctor relationship is easier to establish and thorough examinations and investigations are immediately accessible. Patients who suspect they will receive a teleophthalmology appointment might feel less optimistic about seeking help and therefore delay presentation. Whereas ophthalmologists might feel uncertain about their diagnosis made via teleophthalmology and reluctant to lose time through additional software training and technical issues. The security of personal health data sent via teleophthalmology is also a concern. Artificial intelligence is capable of playing an enormous role in triaging and diagnosing teleophthalmology patients. However, better centralisation of digital patient data is needed to accelerate this process. At the LOCSU National Optical Conference 2022, the focus on developing a telemedicine support service that is shared across an area to provide remote assistance to eyecare professionals with consulting, testing, reviewing, diagnosing and referring was discussed [101]. A centralised facility as such would provide answers to many of the issues associated with telemedicine. However, successful implementation of this will be difficult and requires significant investment. With regards to reducing emissions from medication deliveries, the key obstacle is the financial cost incurred by pharmacies who electrify their delivery vehicles and implement delivery management software. As for serum eye drops, cutting travelling-related emissions has its own unique difficulties. It is very difficult for NHSBT to coordinate deliveries, as patients live far apart and have varying consumption rates of serum eye drops. Furthermore, expansion of the serum eye drops service would involve strenuous attainment of MHRA specialist licensing for each new facility, substantial investment to establish appropriate facilities and trained staff, coordination issues with patients and potential dilution of quality control.

### Promoting sustainability

Government investment in research and innovation to drive NHS net-zero is severely restricted. With the funding that is available, there is limited consistency in how it is allocated across the UK due to the structural complexity of the NHS. Segregation of internal health systems causes differences in care pathways, best practice guidelines and commissioning of newly approved treatments. It also generates differences in how the net-zero activity of NHS suppliers will be monitored and how contracts will subsequently be awarded. In the coming years, NHS England will begin monitoring emissions from suppliers and their progress towards net-zero. This will inevitably deliver new challenges. One of the difficulties will be ensuring suppliers emissions calculations are accurate. It will also be necessary to ensure that sustainability measures are truly being implemented and that any claims of Carbon offsetting are legitimate. In terms of education and awareness, the high service demand across the NHS means multi-disciplinary healthcare workers have little time to learn about sustainable healthcare. This is evidenced by a survey in which only 27% of NHS staff were aware of the NHS’s net-zero ambitions [102]. This casts doubt on the concept that healthcare workers will be able to educate and influence patients to help achieve NHS net-zero goals. As it stands, only 26% of the general public believe the NHS is contributing to climate change [102]. This suggests there is much work to be done (Table 3).

**Table 3:** Summary of opportunities to improve the environmental sustainability of the NHS dry eye disease care pathway.

Time bound	Strategy
**Short term**	• Independent sector Carbon offsetting • Raising awareness of NHS net-zero through adapting public health strategies
**Mid-term**	• Expansion of recycling schemes like TerraCycle • Eco-friendly cardboards and clearer recycling instructions in secondary packaging • More local delivery of care (community optometry workforce, Primary Eyecare Companies and diagnostic and treatment hubs) • Introducing electric vehicles and delivery management systems (e.g., pharmacies and NHSBT) • Government funding to promote sustainability through innovations and research • Regulating Carbon emissions and sustainability progress of NHS suppliers • Training NHS staff on environmentally sustainable healthcare • Development of teleophthalmology and necessary support services
**Long-term**	• Increased use of multi-dose bottles and alternative drug delivery systems • Adaptation of biodegradable packaging products like the Notpla Pipette to create 100% biodegradable single dose eye drops vials • Improving municipal recycling processes so small items are recycled • Reducing the cost of alternative therapies • Electronic devices harnessing renewable energy and recyclable plastics • Further research to reduce dependence on cold chains and improve environmental sustainability of cold chains • Further research around new eye drops formulations including head-to-head studies to compare efficacy and therapeutic duration in subgroups • Better digital connectivity between community eye services and NHS hospitals • Development of artificial intelligence to help triage and diagnose patients • Expansion of NHSBT facilities

## Conclusion

To achieve net-zero in the NHS dry eye disease care pathway, all parties involved must contribute. The UK Government, national and regional NHS health systems, healthcare governing bodies, universities, independent sector companies, healthcare professionals and patients all have a duty to minimise environmental harm and promote good health. It is crucially important to raise awareness of NHS net-zero in these cohorts and to emphasise the collective effort required to reach the current targets. There are numerous opportunities to improve environmental sustainability of the dry eye disease care pathway that can be explored without jeopardising the safety of patients or the financial position of organisations. There will undoubtedly be challenges along the way. It is therefore essential to act now, minimise the environmental damage and protect the wellbeing of current and future generations.
